# Time to Rethink ICS Dosing in Pursuit of Asthma Clinical Remission: High-Dose ICS/LABA May Restore Short-Term Asthma Control When Medium Doses Fail

**DOI:** 10.3390/jcm15145462

**Published:** 2026-07-13

**Authors:** Marta Kołacińska-Flont, Izabela Kupryś-Lipińska, Maciej Wojakiewicz, Tomasz Dębowski, Piotr Kuna

**Affiliations:** 1 Department of Internal Medicine, Asthma and Allergy, Medical University of Lodz, 90-153 Lodz, Poland; piotr.kuna@umed.lodz.pl (P.K.); 2 Medical Department, Chiesi Poland Sp. z o.o., 02-305 Warszawa, Poland; m.wojakiewicz@chiesi.com (M.W.); t.debowski@chiesi.com (T.D.)

**Keywords:** adherence, asthma control, asthma, extra-fine, inhalation technique

## Abstract

**Background/Objectives**: Asthma therapy aims to achieve asthma control, defined as reduced or absent symptoms, no exacerbations and a minimized risk of future untoward outcomes imposed by either the disease or drug side effects. The study aimed to assess the effectiveness and general clinical safety of shifting from medium- to high-dose BDP/FF in a fixed-dose pMDI extra-fine combination as maintenance therapy in patients with poor asthma control despite regular use of a medium-dose fixed formulation of BDP/FF in a real-world setting. **Methods**: BREATH was a multicenter, noninterventional, prospective, real-world based observational study enrolling 93 patients with poor asthma control (ACQ-5 ≥ 1.5) and not meeting Global Initiative for Asthma (GINA) clinical criteria for asthma control despite regular medium-dose BDP/FF use, for whom the treating physician decided to shift to regular high-dose BDP/FF and who were followed for 12 weeks at monthly visits. Adherence/compliance with inhaler therapy, asthma exacerbations and asthma-related emergency visits/hospitalizations were monitored. **Results**: The overall asthma control in the study group improved significantly and progressively, with the mean ACQ-5 score decreasing from 2.8 (SD 0.7) to 0.7 (SD 0.7) points. Visit-to-visit ACQ-5 reductions were significant up to 8 weeks after study entry. The percentage of patients who demonstrated clinically significant improvement in the ACQ-5 score reached 94.4%, with 61.8% fulfilling the ACQ-5 criteria for controlled asthma (58.4% for the GINA clinical criteria). **Conclusions**: Shifting from medium to high doses of BDP/FF in a fixed-dose combination of pMDI and extra-fine BDP/FF in patients who remain symptomatic despite regular medium-dose BDP/FF therapy may improve short-term asthma control.

## 1. Introduction

Asthma is a chronic inflammatory disease of the airways characterized by a history of wheezing, dyspnea, cough, and chest tightness. The goal of asthma therapy is to achieve control of current symptoms, prevent exacerbations and minimize future health hazards. Current control of asthma allows the maintenance of normal activity levels and good quality of life, while the minimization of future risk significantly reduces the likelihood of complications associated with both the disease itself and the side effects of the drugs used for its treatment [1]. Therefore, each step of asthma therapy should be optimized rather than maximized [2].

Despite considerable improvements in the understanding of the underlying mechanism and the availability of novel effective treatments, the prevalence of suboptimal or poor asthma control in real-world settings remains high [1,3,4,5]. The introduction of biologics has revolutionized asthma management, offering excellent treatment results that were unseen with previous therapies [1]. The success of biologics paved the way for the concept of asthma remission as the ultimate treatment goal. Asthma remission has been defined as a prolonged period with minimal or absent symptoms, absent exacerbations, optimized or stable lung function, and reduced or no need for rescue medication, allowing individuals to live a more active and less symptomatic life [1]. Although the term “asthma remission” is usually used in the context of severe asthma and biologics, the GINA report broadens the perspective, indicating that the concept can be applied to asthma of any severity and all types of treatment modalities (including inhaler therapy optimization, the introduction of specific allergen immunotherapy or proper comorbidity management) that lead to a reduction in the daily disease burden [1]. A recent report by Canonica et al. [6] provided surprising data indicating that the effectiveness of inhaled therapy (ICSs, ICSs/LABAs, ICSs/LABAs/LAMAs, and ICSs/LAMAs) in achieving complete and partial asthma remission might be similar to that achieved with biologics. The high cost of biological therapies limits their availability in most public healthcare plans [2]. Therefore, it is especially important to make the most of each step of the asthma management ladder [1,2].

The control of symptoms is considered a key aspect of clinical asthma remission. However, the prevalence of asthma control in daily clinical practice is significantly lower than that reported in many randomized controlled clinical trials, where it reaches 70% of the population surveyed [3,4,5]. Factors affecting the effectiveness of asthma treatment in the real world include incorrect diagnosis, poor inhalation techniques, low patient satisfaction, poor therapy adherence/compliance, unaddressed modifiable risk factors (i.e., irritant exposure and obesity) and overlapping comorbidities [1,5]. Poor asthma control is associated with an increased risk of exacerbation, deterioration of health, reduced quality of life, increased use of healthcare resources and reduced productivity [7,8]. Large-scale European observational studies have shown a high prevalence of poor asthma control, frequent exacerbations, systemic steroid use and asthma-related health care resource utilization [9,10].

The course of asthma is variable, with a fluctuating intensity of symptoms [11,12]. The therapeutic effect of anti-inflammatory asthma control medications such as ICSs develops within days, but it takes approximately 3 to 4 months for the improvement to become clinically evident [1,13]. Previous studies assessing the effectiveness and safety of ICSs as monotherapy in patients with asthma have shown that increasing the ICS dose beyond the medium dose does not provide additional clinical benefit while increasing the incidence of systemic side effects [14]. Therefore, the conventional incremental classification of ICS doses into low, medium, and high categories has recently been challenged [15]. Consequently, in a recent GINA report, fixed combinations of ICSs/LABAs with a high-dose ICS as maintenance therapy in patients with asthma are recommended only on a trial basis in an alternative track [1]. However, most of the research on the effectiveness and safety of ICSs has been performed with fluticasone propionate, budesonide and the standard particle dipropionate beclomethasone, and it is not certain that these results hold true with other ICSs, especially considering the individual variability in ICS responses. A study comparing the dose–response effect on airway hyperresponsiveness and adrenal axis suppression for escalating doses of inhaled fluticasone furoate, fluticasone propionate and budesonide revealed substantial differences in their therapeutic index and questioned the class effect of the group [16]. Evidence from clinical trials with medium- to high-dose fixed combinations of ICSs/LABAs has shown that high-dose ICSs in fixed ICS/LABA combinations significantly improve asthma control while maintaining a favorable safety profile [17,18,19]. The study aimed to assess the effectiveness and safety of shifting from medium- to high-dose BDP/FF in a fixed-dose pMDI extra-fine combination as maintenance therapy in patients with poor asthma control despite regular use of a medium-dose fixed formulation of BDP/FF in a real-world setting.

## 2. Materials and Methods

### 2.1. Study Design

BREATH was a prospective, multicenter, open-label, noninterventional study that included 98 patients who were recruited from allergology/pulmonary practices in Poland. Patients were followed for a total of 12 weeks, with four visits held at 4-week intervals. The medicinal product Fostex^®^ (beclometasone dipropionate/formoterol fumarate dihydrate, 200 μg/6 μg per actuation; pressurized metered dose inhaler (pMDI), Chiesi Farmaceutici S.p.A., Parma, Italy) was prescribed in the usual manner within the routine of medical practice and in accordance with the summary of product characteristics (SmPCs). The decision to enroll a patient in the trial was made independently from the decision to initiate treatment with the high-dose BDP/FF extrafine pMDI therapy. In accordance with the SmPCs of the used BDP/FF formulation (BPD/FF 177.1/5.1 μg pMDI), the recommended dose was two inhalations twice daily, with a maximum daily dose of 4 inhalations per day. No additional diagnostic or monitoring procedures beyond those of routine medical practice were applied.

### 2.2. Inclusion Criteria

- Informed consent expressed in writing by the patient,
- Age ≥ 18 years,
- Confirmed diagnosis of asthma for at least 12 months,
- >3 months use of an average dose of BDP/FF 100/6 (87.5/5 delivered dose), 2 inhalations b.i.d.,
- Treating physician’s decision to increase the dose of ICS to BDP/FF 200/6 (177.1/5.1 delivered dose) b.i.d. because of poor asthma control (ACQ ≥ 1.5).

### 2.3. Exclusion Criteria

- Use of ICSs and LABAs in separate inhalers,
- Use of ICSs at a high dose,
- Incorrect inhalation technique,
- Current participation in a clinical trial,
- Pregnancy or breastfeeding,
- Exacerbation of comorbid conditions.

### 2.4. Study Population and Study Flow, Concomitant Treatment, Exposure to Environmental Factors Affecting Asthma Control

The study began on 31 January 2020 (the first visit of the first patient) and lasted until 27 March 2023 (the last visit of the last participant). A total of 98 patients were screened for study participation at Visit 1, 93 of whom fulfilled the study criteria and were eventually included in the study. Ninety participants completed Visit 2 and 89 completed Visit 3 and Visit 4 (95.7% of the baseline population). In all dropped-out cases, loss to follow-up was due to the SARS-CoV-2 pandemic and restrictions imposed by the epidemiologic situation. With regard to concomitant treatments, no planned changes in asthma-related management other than escalation of maintenance ICS/LABA therapy were permitted during the study period. Similarly, no modifications to the management of chronic comorbid conditions were introduced as part of the study protocol. Patients were required to have stable comorbid conditions at study entry, and no substantial changes in concomitant therapy were permitted. Participants were expected to maintain their usual environmental exposures throughout the observation period. These methodological details have now been clarified in the revised manuscript. The study population demographics and asthma characteristics are presented in Table 1 and Table 2.

### 2.5. Primary Outcome Measures

Improvement in asthma control:

- Mean/median change in the ACQ-5 score at each visit,
- Number of participants who achieved clinically significant improvement in the ACQ-5 score/ACQ-5-defined asthma control at each visit.

### 2.6. Secondary Outcome Measures

Assessment of:

- Rate of improvement in asthma control,
- Number of participants who achieved asthma control according to the 2019 GINA criteria,
- Inhaler therapy adherence/compliance,
- Adverse events (asthma exacerbations, asthma-related hospitalizations and emergency department visits).

### 2.7. Statistical Analysis

Statistical analyses were performed by Biostat Sp. z o. o. in accordance with the ICH E3 and E9 guidelines and standard statistical procedures of Biostat Sp. z o. o.

The R statistical package (version 4.0 or later, R Development Core Team) was used in accordance with the standards of BioStat Sp. z o. o. (PS11/2022 SOP “Data analysis”). *p* values ≥0.001 were reported to 3 decimal places. *p* values less than 0.001 were reported as <0.001. The mean, standard deviation, and any statistics other than quantiles were reported to one more decimal place than the original data. Quantiles, such as medians or minima and maxima, were reported to the same number of decimal places as the original data. Parameter estimates that were not on the same scale as the raw data (e.g., regression coefficients) were rounded to 3 significant figures. The 95% confidence intervals (CIs) for proportions were calculated using the Wilson method with Yeates continuity correction using the prop. test function of R statistical software version R 4.3.0 and R 4.4.1 [20]. Changes in the ACQ-5 score and TAI-10 score were assessed using a mixed model for repeated measures (MMRM), and differences were determined by least squares linear regression analysis. To determine the incidence of adverse events, the exact method (Clopper and Pearson 1934 [21]) implemented in the binom test function was used to calculate confidence intervals. With respect to the incidence rates of exacerbations, hospitalizations, and ED visits, the exact method implemented by the Poisson test function in R was used (Jovanovic and Zalenski 1997 [22]; Agresti and Coull 1998 [23]).

### 2.8. Ethics Board Approval

The study protocol was approved by the local ethics committee on 15 October 2019 (project number RNN/365/19/KE).

### 2.9. Methods for Assessing Asthma Control

*Asthma control questionnaire (ACQ)*

The (ACQ-5) is a validated numerical tool for assessing current asthma control. It is composed of five symptom-related questions. Symptoms are retrospectively assessed within the week preceding evaluation. The GINA recommends the use of a shorter version of the tool (i.e., ACQ-5 instead of the original ACQ).

Asthma control categories according to the ACQ-5 score:

≤0.75 controlled,

>0.75 to <1.5 partially controlled,

≥1.5 uncontrolled.

The clinically significant change in the ACQ was set at 0.5.

*GINA 2019 categories for controlled/partially controlled/uncontrolled asthma*

The GINA 2019 categories of controlled/partially controlled and uncontrolled asthma in the preceding 4 weeks are based on the following aspects:

- Asthma daytime symptoms present > 2×/week,
- Any night waking due to asthma,
- SABA reliever for daytime symptoms used > 2×/week,
- Any activity limitation due to asthma.

If none of these are present, then asthma is defined as well controlled; if 1–2 are present, then asthma is partially controlled, and it is uncontrolled if 3–4 are present.

### 2.10. Methods of Assessing Inhaler Therapy Technique and Inhaler Therapy Adherence/Compliance

Inhaler technique was assessed at the baseline visit (V1) by the treating physician through direct observation of the patient using their own inhaler device. Assessment was performed using a standardized checklist based on the recommended pMDI inhalation steps to ensure that all critical inhalation steps were correctly executed. Patients demonstrating incorrect inhalation technique were excluded from study participation.

No inhaler technique training was provided during the study, and inhaler technique was not reassessed at follow-up visits. The Test of Adherence to Inhalers (TAI) is a questionnaire for patients with asthma or COPD that identifies patients with low adherence to inhaler therapy. It determines the degree of adherence as good, intermediate or poor and classifies patients’ type or pattern of noncompliance as sporadic, deliberate or unconscious.

TAI-10 interpretation:

50 good adherence,

46–49 moderate adherence,

≤45 poor adherence.

## 3. Results

### 3.1. Primary Outcomes

#### 3.1.1. Change in Asthma Control (ACQ-5 Score Reduction)

**Figure 1:**
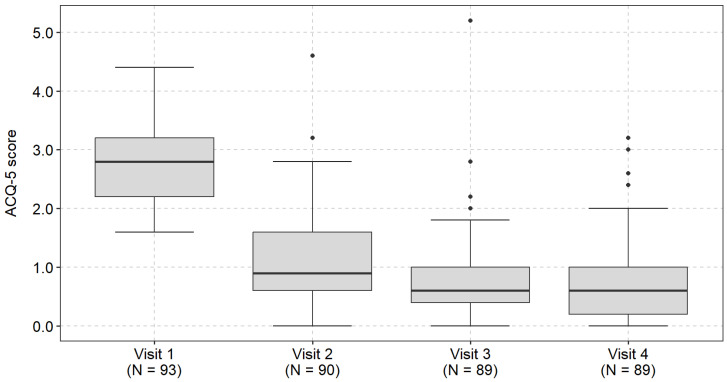
ACQ-5 numerical scores at 4-week intervals throughout the study. N, number of participants.

The mean (and median) ACQ-5 scores decreased from 2.8 (2.8) at V1 to 1.2 (0.9) at V2, 0.8 (0.6) at V3 and ultimately 0.7 (0.6) at V4 (Table 3, Figure 1).

#### 3.1.2. Changes in ACQ-5 Scores vs. Baseline Scores

**Figure 2:**
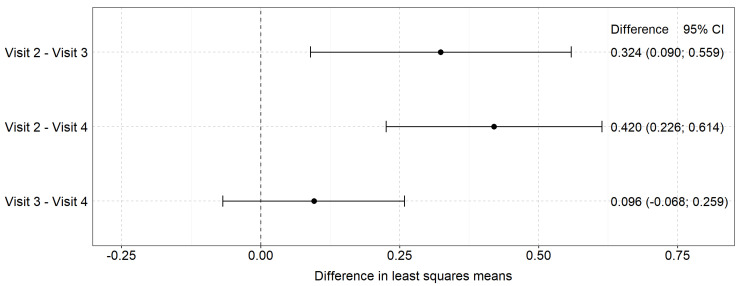
Differences in estimated marginal means (EMMs) for ACQ-5 scores across the visits. EMMs and their respective 95% confidence intervals were estimated using a mixed model for repeated measures (MMRM).

The mean reductions in the ACQ-5 score vs. the baseline score were −1.6 points (SD = 1.0), –1.9 (SD 1.0) and –2.0 (SD 0.9) at V2, V3 and V4, respectively. The most pronounced decline from baseline was recorded at V3 (–4.2 points) (Table 4). Reductions in ACQ-5 scores at each study visit vs. baseline were significant (Table 5). The intervisit differences were significant for the V2 vs. V3 and V2 vs. V4 comparisons but not for the V3 vs. V4 comparison. The reduction in the ACQ-5 score peaked at V3 and did not improve further. This finding indicates that the majority of clinical improvement occurred by Visit 3, with symptom reduction plateauing thereafter (Figure 2). The proportion of patients demonstrating a clinically significant improvement in the ACQ-5 score (change in ACQ-5 score ≥0.5) increased progressively, reaching 85.6%, 93.3% and 94.4% at V2, V3 and V4, respectively.

#### 3.1.3. Number of Participants Who Achieved Clinical Asthma Control

**Figure 3:**
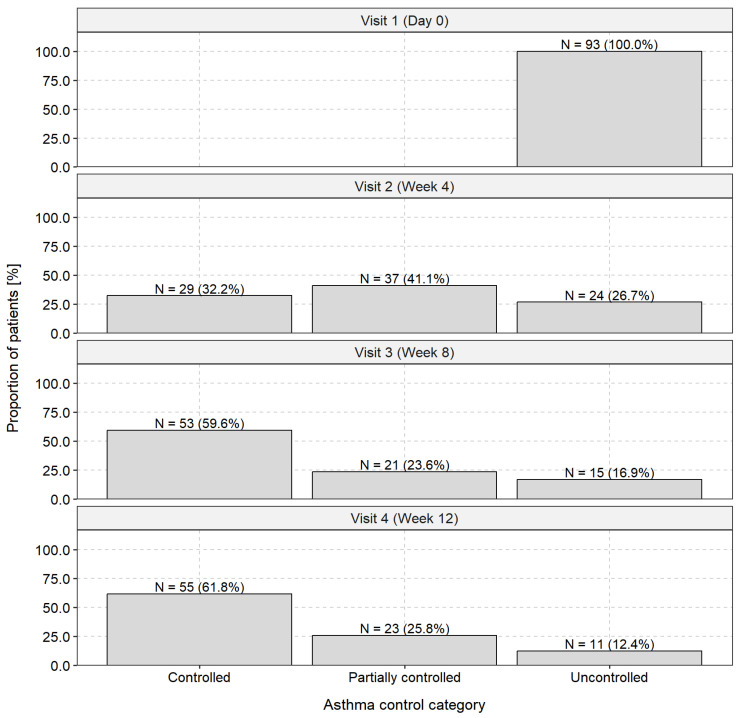
Proportion of patients with uncontrolled, controlled and partially controlled asthma according to the ACQ score. N, number of participants.

At baseline, 100% of the study participants had clinically uncontrolled asthma (ACQ-5 ≥ 1.5). The proportion of patients who achieved clinical control of asthma (ACQ-5 ≤ 0.75) was 32.2%, 59.6% and 61.8% at V2, V3 and V4, respectively. Conversely, the percentage of patients with uncontrolled asthma steadily declined, ultimately reaching 12.4%. (Figure 3).

### 3.2. Secondary Outcome Measures

#### 3.2.1. Asthma Control According to the GINA Clinical Criteria

**Figure 4:**
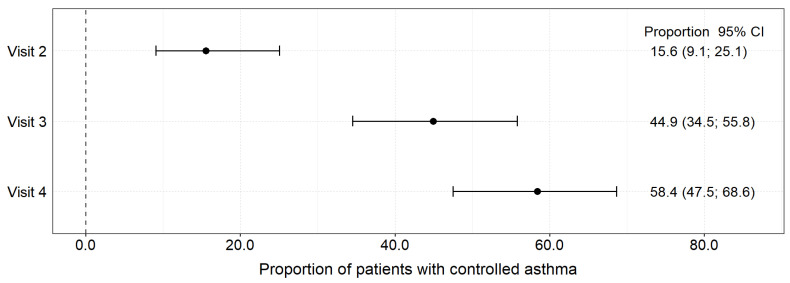
Comparison of the proportion of patients with clinically controlled asthma according to the GINA clinical criteria at 4-week follow-up visits. Changes in the proportion of patients with controlled asthma over time were assessed using a one-sample test for proportions based on a chi-square approximation with Yates’ continuity correction, as implemented in the R statistical environment (prop. test function). For each follow-up visit, the observed proportion of patients with controlled asthma was compared with the baseline proportion. Statistical significance was defined as a two-sided *p*-value < 0.05.

The GINA clinical criteria for asthma control consider a longer retrospective period (4 weeks) than the ACQ-5 score does (7 days). At baseline, none of the participants met the GINA criteria for controlled asthma, while at the end of the study, 52 participants (58.4%) fulfilled the criteria for controlled asthma (Figure 4).

#### 3.2.2. Comparison of Categorization Outcomes: ACQ-5 vs. GINA 2019 Criteria

In both classification methods, a progressive shift in patients from the uncontrolled asthma category to the partially controlled and controlled categories was observed. The Cohen’s kappa coefficients were 0.34, 0.37 and 0.50 for V2, V3, and V4, respectively, indicating low but steadily increasing agreement between the two assessment methods.

#### 3.2.3. Assessment of Inhalation Therapy Adherence/Compliance and Types of Noncompliant Behaviors in the Study Population

**Figure 5:**
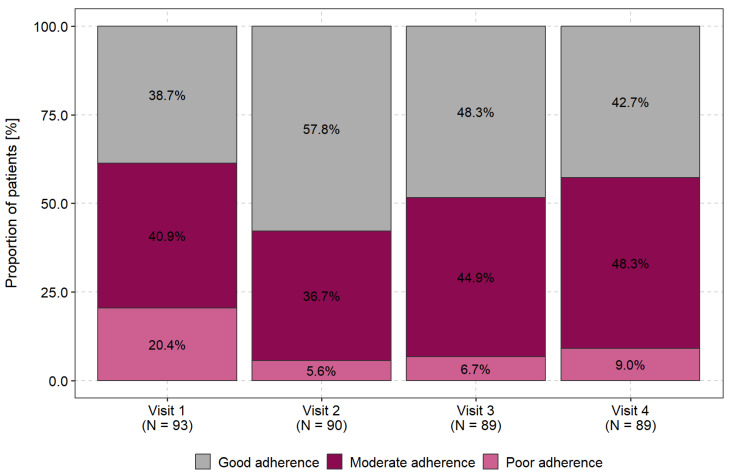
Adherence to inhaler therapy according to the TAI-10. N, number of participants.

The TAI-10 results indicated that at study entry (V1), 36 patients (38.7%) demonstrated good, 38 (40.9%) demonstrated intermediate and 19 (20.4%) demonstrated poor adherence to inhaler therapy. Although there was an improvement in adherence rates at V2, the overall results at the end of the study did not differ from those at baseline, with 38 patients (42.7%) demonstrating good adherence, 43 patients (48.3%) demonstrating intermediate adherence and 8 patients (9%) showing poor adherence (Figure 5).

Greater adherence at baseline was associated with a greater decrease in TAI-10 scores over time, implying that patients with initially high adherence tend to show some reduction in adherence at follow-up visits. For the intervisit changes in the TAI-10, only V2 and V4 were statistically significant (Table 6).

#### 3.2.4. Predictors of Treatment Response

**Figure 6:**
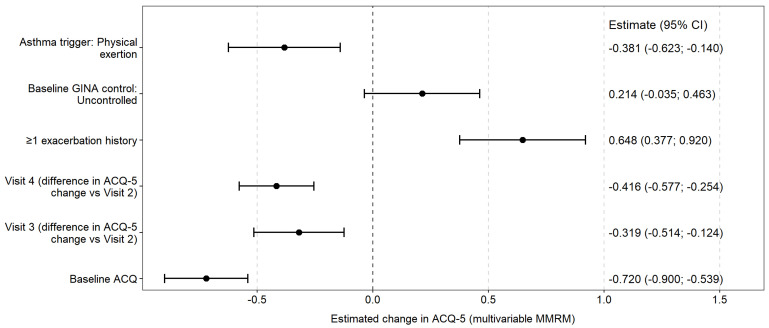
Multivariable MMRM analysis of factors associated with change in ACQ-5. Note: Each point represents the estimated effect from multivariable MMRM models with corresponding 95% confidence intervals. The dashed vertical line indicates no effect. Positive values indicate an increase in ACQ-5 (worsening asthma control), while negative values indicate a decrease (improvement in asthma control).

The following baseline characteristics were included in the analyses of predictors of treatment response: asthma duration, history of exacerbations, body mass index (BMI), smoking status, smoking exposure (pack-years), baseline GINA symptom control category, treatment adherence (TAI-10), and patient-reported asthma triggers, including perennial and seasonal allergens, infections, physical exertion, exposure to cold air, tobacco smoke, odors, speaking/laughing, and other triggers. To identify independent predictors of treatment response, a multivariable mixed model for repeated measures (MMRM) was performed subsequently. Higher baseline ACQ-5 scores and physical exertion as a trigger of asthma symptoms were independently associated with greater improvement in asthma control during follow-up (*p* < 0.001 and *p* = 0.002, respectively), whereas a history of at least one asthma exacerbation was independently associated with a smaller improvement in ACQ-5 (*p* < 0.001) (Table 7, Figure 6).

#### 3.2.5. Safety Outcomes: Incidence of Severe Asthma Exacerbations, Hospitalizations and ED Visits Because of Asthma

Throughout the entire duration of the study, a total of 4 asthma exacerbations (4.4%) were recorded (incidence rate 0.04 exacerbations/100 patient-weeks). None of the study participants required hospitalization or emergency department (ED) visits because of asthma.

## 4. Discussion

Asthma remission is defined as a prolonged period of disease stability characterized by the absence of symptoms, no exacerbations, stable lung function, and minimal or no use of systemic corticosteroids [1,6,24,25,26]. The remission of asthma can be classified into clinical remission (sometimes called partial remission), where symptoms are controlled and lung function is preserved, and complete remission, which includes the resolution of airway inflammation. Achieving remission is an important goal in asthma management because it offers long-term benefits beyond symptom control. Patients who achieve remission experience fewer exacerbations, improved quality of life, lower reliance on medications, and reduced healthcare costs [24,25,26]. While remission is often discussed in the context of severe asthma and biologic therapy, the concept is applicable to all patients with asthma, regardless of severity or treatment type [1]. Clinical/partial remission can also be induced through the initiation of inhaled corticosteroids (ICSs), combination therapy with long-acting beta-agonists (LABAs)/ICSs, ICS dose escalation, or allergen-specific immunotherapy (AIT) [1,6]. Recently, published results from a cross-sectional study of the Severe Asthma Network in Italy (SANI) provided preliminary evidence that improved inhaled therapy based on ICS/LABA or ICS/LABA/LAMA combined therapy is not inferior to that produced by biologics in terms of achieving asthma remission, which is especially appealing in view of the cost of biologics and their low availability in economically challenged settings [6].

Asthma control is a key component of remission and refers to the degree to which symptoms are minimized while future risks, such as exacerbations and lung function decline, are reduced [1,24,25,26]. The Global Initiative for Asthma (GINA) guidelines categorize asthma control as controlled, partly controlled, and uncontrolled on the basis of daytime and nighttime symptoms, activity limitations, use of reliever medication, lung function, and exacerbation history. Achieving good clinical control is necessary for progressing toward remission, and poor control is associated with an increased risk of asthma exacerbations and increased morbidity and mortality in asthma patients [1,24,25,26]. The five-question Asthma Control Questionnaire (ACQ-5) is among the validated numerical tools for asthma control assessment and is recommended for tracking treatment effectiveness and adjusting therapy accordingly [1,27].

Previous studies have shown that although the intensity of anti-inflammatory therapy is crucial for achieving symptom control in patients with asthma, increasing the ICS dose beyond the medium range not only does not improve outcomes but is also associated with an increased incidence of side effects [27,28,29,30]. The dose–response relationship of inhaled corticosteroids (ICSs) in asthma treatment has been extensively studied, and evidence suggests that most of the therapeutic benefit is achieved at low-to-medium doses [27,28,29,30]. According to systematic reviews and meta-analyses, 80–90% of the maximum effect on asthma control, lung function, and exacerbation reduction is attained at doses classified as “low” or “medium” in the traditional guidelines [14,15]. In general, higher doses of ICSs do not provide proportionally greater improvements in lung function, asthma symptoms, or exacerbation reduction but are associated with a greater risk of systemic side effects, including adrenal insufficiency, osteoporosis, and diabetes. This challenges the conventional classification of ICS doses into low, medium, and high categories, as the increases between these levels do not necessarily translate into clinically significant benefits [15]. However, this view is based on evidence derived mainly from studies with fluticasone propionate, beclomethasone dipropionate standard particles and budesonide in monotherapy, most often delivered from dry powder inhalers [28,29,30]. Significant interpersonal variability in response to ICSs has been observed, suggesting that some patients (mainly those with unsatisfactory responses to medium-dose ICSs) might benefit from higher ICS dosing [14]. Moreover, differences in the therapeutic indices between various ICSs and different responses to various formulations of the same molecule (i.e., fluticasone propionate vs. fluticasone furoate) have been observed [24]. Interclass variability in the potency of ICSs should not be surprising considering the established differences in the pharmacokinetics, pharmacodynamics and receptor binding capacity of the molecules in this group of drugs [31]. In the case of topical steroids, the fact that formulations, concentrations and vehicles are all factors affecting drug potency, leading to differences in clinical effectiveness and safety, has become part of the treatment guidelines and incorporated into everyday practice [32]. Considering the route of administration and the need to cross the epithelial barrier in a precise spot, i.e., small airways, to achieve the desired therapeutic effect, it seems reasonable to regard ICSs from a similar perspective as topical drugs, considering the effects of the type of inhaler device and molecular size on lung deposition as well. Extra-fine formulations of the BDP/FF pMDIs achieve high rates of intrathoracic drug deposition, with peripheral lung distributions of more than 30% (depending on the flow rate) [33]. Determination of whether these results affect clinical ICS effectiveness requires further studies, as the evidence published thus far is inconsistent [34,35].

A UK database study found no evidence that a step-up to high-dose ICSs is effective in preventing future asthma exacerbations [36]. Given the large sample size, length of follow-up, amount of clinical data included in the analysis and the real-world setting, the study provides valuable evidence. However, this study has several limitations including its retrospective design, its diagnosis of asthma based on codes assigned to the visits (i.e., patients with COPD were included, but a sensitivity analysis excluding these patients was performed) and a lack of data on asthma clinical control. Moreover, the analysis included all prescriptions for ICSs, either alone or in combination with inhalers, and most patients on high-dose ICSs were prescribed fluticasone propionate. Therefore, the presented outcomes cannot be simply pooled over the whole ICS class and ICS/LABA fixed-dose formulations. Furthermore, the study did not investigate symptom reduction, as reducing the everyday disease burden is considered a cornerstone of clinical remission of asthma.

Whether the initial findings on the flat dose–effect curve hold true for different ICS doses within fixed-dose ICS/LABA combination therapies remains undetermined. Evidence comes mainly from clinical trials; however, only a few have explored the effects of different ICS doses within fixed ICS/LABA formulations on the clinical control of asthma. The landmark for the use of ICSs/LABAs as maintenance therapy in asthma studies, the Gaining Optimal Asthma ControL Study, which was designed to compare the efficacy of increasing doses of fluticasone propionate alone or in combination with the long-acting 2-agonist salmeterol in terms of achieving asthma control, revealed that more patients on maintenance treatment at higher doses achieve this goal [34,35]. Considering that the addition of LABAs allows disease control to be achieved with lower ICS dosages, increasing the ICS component dose within these formulations might offer additional advantages. Data from clinical trials with ICSs/LABAs at high doses that compared them to medium-dose ICSs/LABAs or a high-dose ICS in monotherapy did not raise any safety concerns [16,17,18,37,38]. To date, there is a paucity of evidence on the effect of stepping up from medium- to high-dose ICSs in the same fixed-dose combination of ICSs/LABAs on clinical control of asthma in real-world studies.

Our results provide evidence that shifting from medium- to high-dose extra-fine BDP/FF pMDI can improve clinical asthma control and is safe and well tolerated. Among the 100% of patients with uncontrolled asthma at study entry, 61.8% had controlled asthma, 25.8% had partially controlled asthma, and only 12.4% remained in the uncontrolled ACQ-5 category at the final visit. Similar shifts in asthma control categories were seen in the GINA-based clinical categorization, with 52 patients (58.4%) ultimately falling into the controlled asthma category. Although significant decreases in the ACQ-5 score were already observed 4 weeks after increasing the ICS dose, the most prominent clinical improvement was recorded at 8 weeks, and the score did not improve any further, indicating that the maximal benefit of the shift in the ICS dose was achieved within 8 weeks of treatment intensification. Therefore, the optimal waiting time for clinical benefit after increasing the ICS dose in an ICS/LABA fixed combination is between 4 and 8 weeks and should not be prolonged further. This time span is much shorter than the time proposed by the GINA for a trial of high-dose ICSs (3–6 months) [1]. A shorter exposure time decreases the risk of hypothetical side effects; therefore, it might encourage asthma-treating physicians to perform such trials of high-dose ICSs/LABAs. Beyond 8 weeks, in patients still experiencing bothersome daily asthma symptoms, other means of improving asthma control should be considered, e.g., the addition of a LAMA, the introduction of allergen immunotherapy if appropriate or referral for biologics if indicated and available [1].

In addition to the type and intensity of anti-inflammatory therapy, several other factors that may affect asthma control and inhaler therapy adherence are among the most important. Therapy adherence is defined as the degree to which a patient follows prescribed treatment recommendations, and in patients with asthma, this refers particularly to controller therapy, i.e., inhaled corticosteroids [1,39]. High adherence rates to ICSs are associated with better asthma control, fewer exacerbations, fewer preventable hospitalizations and lower mortality in asthma patients. Increasing adherence by just 25% can reduce asthma-related hospitalizations by 31–39%, demonstrating the significant impact of adherence on healthcare costs [40,41,42]. Nonadherence to asthma medications leads to higher direct healthcare costs (hospital admissions and emergency visits) and indirect costs (lost productivity) [43]. Types of inhalers differ in terms of the skills required to operate them [1]. Pressured metered dose inhalers allow for extra-fine particle delivery to small airways but are particularly challenging in terms of the proper inhaler technique [44]. Although this type of inhaler does not require strong and forceful inhalation, making it suitable even for patients with severe airflow limitation, it does require proper hand-breath coordination [44]. Incorrect inhalation techniques lead to poor drug deposition in the lungs, reduced therapeutic effectiveness, and increased exacerbation risk, affecting patient satisfaction with therapy and impacting therapy adherence [45]. Patients with poor adherence are excluded from clinical trials, which might explain the differences in rates of asthma control reported with inhaler treatment in clinical trial results vs. those achieved in everyday practice. To account for this, our study included an evaluation of the proper inhaler technique and adherence to inhaler therapy with the use of validated TAI-10 scores [46]. While overall adherence in our study remained high, both intentional and unintentional noncompliance exhibited dynamic patterns over time, highlighting the need for ongoing adherence/compliance monitoring and support in asthma management. TAI-10 scores improved during the early phase of follow-up and subsequently declined, although adherence remained slightly higher at the final visit compared with baseline. This pattern may reflect a behavioral effect associated with study participation and increased attention to inhaled therapy during the enrolment process. To minimize the potential impact of inhaler technique refinement on study outcomes, no inhaler technique training was provided at enrolment or during follow-up visits, and inhaler technique was not reassessed after baseline. Nevertheless, we acknowledge that participation in a prospective observational study and repeated assessment of treatment adherence may have contributed to improved treatment-related behaviors and, consequently, to some of the observed clinical benefits. A multivariable analysis was performed to identify predictors of treatment response. The analysis included the following baseline characteristics: asthma duration, history of exacerbations, body mass index (BMI), smoking status, smoking exposure (pack-years), baseline GINA symptom control category, treatment adherence (TAI-10), and patient-reported asthma triggers, including perennial and seasonal allergens, infections, physical exertion, exposure to cold air, tobacco smoke, odors, speaking/laughing, and other triggers. The results suggest that escalation to high-dose ICS within a fixed-dose ICS/LABA combination may be particularly beneficial in patients with poorer baseline asthma control (high ACQ-5, physical exertion as asthma trigger), whereas patients with a history of asthma exacerbations may experience a smaller improvement in asthma control with this strategy alone.

### 4.1. Study Strengths

The BREATH study was specifically designed to evaluate the effectiveness of inhaled therapy under real-world clinical conditions. A major strength of the study was the comprehensive assessment of factors that critically influence treatment effectiveness but are frequently underreported in large observational studies, including inhaler technique, treatment adherence, and patient satisfaction with therapy. Participants were recruited from tertiary referral centers specializing in pulmonology and allergology, providing advanced asthma diagnostics and management, including biological therapies. This approach enhanced the accuracy of asthma diagnosis and ensured careful assessment of treatment response throughout the study period. Consistent with its real-world design, the study focused on clinically meaningful outcomes, particularly asthma control and symptom burden. Although advanced assessments of airway inflammation and inflammatory endotypes were not performed, such investigations remain unavailable in many routine clinical settings. Consequently, the study reflects circumstances commonly encountered in everyday asthma practice and may therefore have high external validity. The findings suggest that increasing the ICS dose within the same fixed-dose ICS/LABA combination inhaler may provide clinically meaningful improvements in asthma control in selected patients who remain uncontrolled despite medium-dose maintenance therapy. To our knowledge, this is among the first real-world studies evaluating the clinical impact of escalating the corticosteroid dose within the same fixed-combination ICS/LABA inhaler without changing the inhalation device. The results further suggest that most of the clinical benefit associated with treatment escalation was observed within the first 8 weeks of therapy. Although this observation requires confirmation in larger controlled studies, it may indicate that treatment response can be assessed earlier than the 3–6-month period often considered for therapeutic trials of high-dose ICS-containing regimens. If confirmed, such findings could help inform clinical decision-making regarding the timing of alternative treatment escalation strategies, including triple therapy or biologic treatment. Finally, the observed variability in treatment response highlights the need for a more individualized approach to inhaled therapy selection. Future research should further explore how factors such as the corticosteroid molecule, aerosol formulation, particle size, inhaler device, and dosing strategy influence clinical outcomes, with the aim of supporting more personalized management of asthma.

### 4.2. Study Limitations

Several limitations of the present study should be acknowledged. First, the sample size was relatively small, and no comparator group was included. Second, lung function parameters and biomarkers of type 2 inflammation, such as fractional exhaled nitric oxide (FeNO) and peripheral blood eosinophil counts, were not collected. The study was designed primarily to evaluate the effect of switching to high-dose ICS/LABA therapy on clinical asthma control in a real-world setting rather than to investigate physiological or inflammatory mechanisms underlying treatment response. In addition, the relatively short duration of follow-up limits the assessment to short-term clinical outcomes and does not allow conclusions regarding the long-term effectiveness or safety of treatment intensification. In particular, no objective measures of hypothalamic–pituitary–adrenal (HPA) axis function were included. Therefore, the present study cannot directly address the systemic effects of prolonged exposure to high-dose inhaled corticosteroids. However, it should be noted that in the pivotal clinical development program of extrafine BDP/FF 200/6 μg, treatment durations of up to 24 weeks were evaluated without evidence of clinically relevant HPA axis suppression. Nevertheless, dedicated long-term studies incorporating objective assessments of adrenal function are warranted.

### 4.3. Future Research Directions

Further studies specifically designed to investigate the role of inhaled therapy optimization in terms of increasing ICS dose within ICS/LABA combination in achieving and maintaining long-term asthma remission are warranted. Ideally, such studies should include direct head-to-head comparisons of different single-inhaler combination regimens, including regular medium-dose ICS/LABA, high-dose ICS/LABA, and medium- or high-dose ICS/LABA/LAMA therapies and a long-term observation period (i.e., 12 months). The ultimate goal of this research would be to facilitate a more individualized approach to inhaled treatment escalation in patients who remain symptomatic despite standard controller therapy. Additional studies evaluating the long-term safety of regular high-dose ICS/LABA treatment are also needed. Particular attention should be given to clinically relevant outcomes, including hypothalamic–pituitary–adrenal axis function, metabolic effects, and growth-related parameters in pediatric populations. While previous reports have raised concerns regarding the safety of prolonged high-dose ICS use, many of these studies were conducted using earlier inhaler formulations and delivery systems. Whether the safety profile of contemporary ICS formulations differs from that of older preparations requires further investigation. The findings of the present study also highlight the need for a better understanding of factors that may influence the clinical effectiveness of inhaled corticosteroids. In addition to dose, these factors may include the corticosteroid molecule itself (e.g., fluticasone furoate, budesonide, or beclomethasone dipropionate), aerosol characteristics (standard versus extrafine particles), and the mode of drug delivery (e.g., dry powder inhalers versus pressurized metered-dose inhalers, with or without spacer devices). Improved characterization of these determinants may help explain interindividual variability in treatment response and support the development of more personalized treatment strategies for patients with asthma.

## 5. Conclusions

This multicenter, noninterventional, prospective observational study provides preliminary real-world based evidence that intensifying anti-inflammatory therapy by shifting from regular medium to high doses of ICSs in a fixed-dose formulation with an extra-fine BDP/FF pMDI may improve short-term clinical asthma control without raising major safety concerns. The maximal improvement in terms of response rate and the degree of improvement in asthma control occurred after 8 weeks of treatment and remained stable thereafter. The study findings suggest that patients with uncontrolled asthma receiving a fixed-dose ICS/LABA formulation with a medium-dose ICS may be offered a trial of regular high-ICS-dose ICS/LABA fixed-dose combination, preferably with extra-fine particles, before escalation to other interventions is considered. An individualized approach to asthma management with careful assessment of factors potentially affecting inhaler therapy effectiveness, i.e., the type of ICS particle and inhaler device, as well as individual response to ICS requiring higher doses, can help fill the gap between inhaler therapy and more costly interventions in the pursuit of long-term asthma remission.

## Figures and Tables

**Table 1:** Baseline demographics of the study population.

Parameter	N = 93
Gender	
Female	60 (64.5%)
Male	33 (35.5%)
Age [years]	
N	93
mean (SD)	52.8 (12.6)
median (Q1–Q3)	53.0 (44.0–64.0)
min–max	25.0–77.0
Education level	
Primary	5 (5.4%)
Vocational	9 (9.7%)
Secondary	39 (41.9%)
Incomplete higher	6 (6.5%)
Higher	34 (36.6%)
Weight [kg]	
N	93
mean (SD)	75.2 (15.1)
median (Q1–Q3)	75.0 (62.0–86.0)
min–max	48.0–112.0
Height [cm]	
N	93
mean (SD)	169.6 (9.4)
median (Q1–Q3)	168.0 (163.0–177.0)
min–max	150.0–195.0
BMI [kg/m^2^]	
N	93
mean (SD)	26.2 (5.1)
median (Q1–Q3)	26.2 (22.3–29.3)
min–max	16.7–41.1
Body weight categorization [1]	
Normal body weight	31 (33.3%)
Overweight	35 (37.6%)
Underweight	7 (7.5%)
Obese	20 (21.5%)
Smoking	
Smoker	4 (4.3%)
Ex-smoker	10 (10.8%)
Nonsmoker	77 (82.8%)
No data	2 (2.2%)
Packyears of smoking for smokers/ex-smokers [2]	
N	14
mean (SD)	10.6 (14.9)
median (Q1–Q3)	6.8 (5.0–7.9)
min–max	1.8–60.0

Descriptions: [1] BMI categories: <18.5—underweight, <18.5–25—normal weight, <25–30—overweight, ≥30 kg/m^2^—obesity. [2] Number of pack-years of smoking: Packyears = (mean number of cigarettes smoked per day/20 × timespan of smoking habit in years. N, number of participants. SD, standard deviation. Min-max, minimal and maximal value.

**Table 2:** Baseline asthma characteristics in the study population.

Parameter	N = 93
Asthma duration in years [1]	
N	93
mean (SD)	16.1 (12.2)
median (Q1–Q3)	12.1 (7.0–19.7)
min–max	1.2–59.1
Factors provoking asthma attacks [2]	
Unidentified	5 (5.4%)
One factor	18 (20.5%)
More than one factor	70 (79.5%)
Perennial allergens	33 (35.5%)
Seasonal allergens	15 (16.1%)
Exposure to intense fragrances	26 (28.0%)
Exertion	64 (68.8%)
Exposure to cold air	38 (40.9%)
Cigarette smoke	26 (28.0%)
Speech, laughter	2 (2.2%)
Infection	45 (48.4%)
Other factors	3 (3.2%)
Number of asthma exacerbations requiring systemic GCS in the preceding 12 months	
No exacerbations	64 (68.8%)
At least one exacerbation	29 (31.2%)
mean (SD)	1.4 (0.6)
median (Q1–Q3)	1.0 (1.0–2.0)
min–max	1.0–3.0
Number of hospitalizations due to asthma in the preceding 12 months [3]	
No hospitalizations	89 (95.7%)
At least one hospitalization	4 (4.3%)
mean (SD)	1.2 (0.5)
median (Q1–Q3)	1.0 (1.0–1.2)
min–max	1.0–2.0
Asthma control according to GINA 2019 clinical categorization	
Partially controlled asthma	39 (41.9%)
Uncontrolled asthma	54 (58.1%)
General state of health in self-assessment	
Excellent (5)	2 (2.2%)
Very good (4)	11 (11.8%)
Good (3)	57 (61.3%)
Average (2)	21 (22.6%)
Poor (1)	2 (2.2%)
General state of health in self-assessment (ordinal variable)	
N	93
mean (SD)	3.1 (0.5)
median (Q1–Q3)	3.0 (3.0–3.0)
min–max	1.0–5.0
Does the patient adhere to the treatment (physician opinion)?	
Yes, at all times	60 (64.5%)
Yes, sporadic nonadherence not affecting overall asthma control in the treating physician opinion	33 (35.5%)
No	0 (0.0%)
Asthma control according to ACQ-5	
N	93
mean (SD)	2.8 (0.7)
median (Q1–Q3)	2.8 (2.2–3.2)
min–max	1.6–4.4

[1] Asthma duration was defined as the time elapsed between the date of the informed consent signature and the date of asthma diagnosis. [2] The values sum to >100% because >1 factor could be selected. [3] Asthma exacerbation defined as per the GINA 2019 definition [1]. N, number of participants. SD, standard deviation. Min-max, minimal and maximal value.

**Table 3:** ACQ-5 scores at 4-week intervals.

Parameter	Visit 1	Visit 2	Visit 3	Visit 4
N	93	90	89	89
ACQ-5 mean score (SD)	2.8 (0.7)	1.2 (0.9)	0.8 (0.8)	0.7 (0.7)
ACQ-5 median score (Q1–Q3)	2.8 (2.2–3.2)	0.9 (0.6–1.6)	0.6 (0.4–1.0)	0.6 (0.2–1.0)
ACQ-5 score min–max	1.6–4.4	0.0–4.6	0.0–5.2	0.0–3.2

N, number of participants. SD, standard deviation. Min-max, minimal and maximal value.

**Table 4:** Changes in the ACQ score from baseline throughout the 12-week study period.

Parameter	V2 (4 Weeks)	V3 (8 Weeks)	V4 (12 Weeks)
N	90	89	89
Mean ACQ reduction (SD) vs. baseline	−1.6 (1.0)	−1.9 (1.0)	−2.0 (0.9)
Median ACQ reduction (Q1–Q3) vs. baseline	−1.8 (−2.4; −1.0)	−2.0 (−2.6; −1.2)	−2.2 (−2.6; −1.6)
Min–max ACQ reduction vs. baseline	−4.0–0.8	−4.2–1.6	−4.0–1.2

SD, standard deviation. Min-max, minimal and maximal value.

**Table 5:** Estimated marginal means (EMMs) of ACQ-5 scores for each visit vs. baseline (V1) and between the visits thereafter (V2 vs. V3 and V3 vs. V4).

Model Estimate	Estimated Marginal Means	95% CI	*p* Value
Visit 2	−1.615	−1.792; −1.438	<0.001
Visit 3	−1.939	−2.108; −1.771	<0.001
Visit 4	−2.035	−2.188; −1.882	<0.001
	Difference in EMMs		
Visit 2 vs. Visit 3	0.324	0.090; 0.559	0.004
Visit 2 vs. Visit 4	0.420	0.226; 0.614	<0.001
Visit 3 vs. Visit 4	0.096	−0.068; 0.259	0.347

EMMs and differences in EMMs and their respective 95% confidence intervals were estimated using a mixed model for repeated measures (MMRM).

**Table 6:** Estimated marginal means (EMMs) of TAI-10 scores for each visit vs. baseline and between the visits thereafter.

Model Coefficient	Estimated Marginal Means	95% CI	*p* Value
Visit 2	1.154	0.799; 1.509	<0.001
Visit 3	0.790	0.382; 1.197	<0.001
Visit 4	0.483	0.026; 0.939	0.038
	Difference in EMMs		
Visit 2–Visit 3	0.364	−0.026; 0.755	0.073
Visit 2–Visit 4	0.671	0.138; 1.204	0.010
Visit 3–Visit 4	0.307	−0.198; 0.811	0.320

EMMs and differences in EMMs and their respective 95% confidence intervals were estimated using a mixed model for repeated measures (MMRM).

**Table 7:** Multivariable MMRM analysis of baseline predictors associated with change in ACQ-5.

Predictor	Estimate	95% CI	*p*-Value
Baseline ACQ	−0.720	−0.900; −0.539	<0.001
Visit 3 (difference in ACQ-5 change vs. Visit 2)	−0.319	−0.514; −0.124	0.002
Visit 4 (difference in ACQ-5 change vs. Visit 2)	−0.416	−0.577; −0.254	<0.001
≥1 exacerbation history	0.648	0.377; 0.920	<0.001
Baseline GINA control: Uncontrolled	0.214	−0.035; 0.463	0.091
Asthma trigger: Physical exertion	−0.381	−0.623; −0.140	0.002

Note: Results are from a multivariable mixed model for repeated measures (MMRM) with change from baseline in ACQ-5 as the dependent variable. The model included baseline ACQ-5, visit, exacerbation history, baseline GINA control category, and physical exertion (asthma trigger) as fixed effects. An unstructured covariance matrix was used to account for within-subject correlations across visits. Estimates represent adjusted associations with change from baseline in ACQ-5. Negative estimates indicate greater improvement in ACQ-5, whereas positive estimates indicate less improvement (or worsening). 95% confidence intervals were derived from model estimates. Statistical significance was assessed at a two-sided α level of 0.05. As a sensitivity analysis, all possible combinations of candidate baseline predictors were evaluated using Akaike’s Information Criterion (AIC). The model presented in Table 2 achieved the lowest AIC value among the candidate models. The coefficients for Visit 3 and Visit 4 represent the model-estimated difference in change in ACQ-5 compared with the reference visit (Visit 2). This means that the model compares the magnitude of change in ACQ-5 at subsequent visits (V3 and V4) with the change observed at Visit 2.

## Data Availability

The data generated and analyzed in the present study are available upon reasonable request from the corresponding author due to concerns regarding privacy and ethical reasons.
